# Biological Significance of microRNA Biomarkers in ALS—Innocent Bystanders or Disease Culprits?

**DOI:** 10.3389/fneur.2019.00578

**Published:** 2019-06-11

**Authors:** Sophie Foggin, Raquel Mesquita-Ribeiro, Federico Dajas-Bailador, Rob Layfield

**Affiliations:** School of Life Sciences, University of Nottingham Medical School, Nottingham, United Kingdom

**Keywords:** microRNA, amyotrophic lateral sclerosis, ALS, MND, biomarker, ALS genes, bioinformatics, neurodegeneration

## Abstract

MicroRNAs (miRNAs) represent potential biomarkers for neurodegenerative disorders including amyotrophic lateral sclerosis (ALS). However, whether expression changes of individual miRNAs are simply an indication of cellular dysfunction and degeneration, or actually promote functional changes in target gene expression relevant to disease pathogenesis, is unclear. Here we used bioinformatics to test the hypothesis that ALS-associated miRNAs exert their effects through targeting genes implicated in disease etiology. We documented deregulated miRNAs identified in studies of ALS patients, noting variations in participants, tissue samples, miRNA detection or quantification methods used, and functional or bioinformatic assessments (if performed). Despite lack of experimental standardization, overlap of many deregulated miRNAs between studies was noted; however, direction of reported expression changes did not always concur. The use of *in silico* predictions of target genes for the most commonly deregulated miRNAs, cross-referenced to a selection of previously identified ALS genes, did not support our hypothesis. Specifically, although deregulated miRNAs were predicted to commonly target ALS genes, random miRNAs gave similar predictions. To further investigate biological patterns in the deregulated miRNAs, we grouped them by tissue source in which they were identified, indicating that for a core of frequently detected miRNAs, blood/plasma/serum may be useful for future profiling experiments. We conclude that *in silico* predictions of gene targets of deregulated ALS miRNAs, at least using currently available algorithms, are unlikely to be sufficient in informing disease pathomechanisms. We advocate experimental functional testing of candidate miRNAs and their predicted targets, propose miRNAs to prioritise, and suggest a concerted move towards protocol standardization for biomarker identification.

## Introduction

MicroRNAs (miRNAs) are small non-coding RNAs, typically 20–22 nucleotides (nt) long, which act as post-transcriptional regulators of gene expression (1). MiRNA seed sequences provide specificity for the 3′ untranslated region (UTR) of target mRNA, leading to mRNA degradation or translational inhibition (2). Around a third of human gene products are regulated by miRNAs (3), being present in both intracellular and extracellular environments and in almost all biological fluids (4, 5). Extracellularly, miRNAs are detected within membrane vesicles and also freely, forming complexes with other macromolecules (6).

Amyotrophic lateral sclerosis (ALS) is characterized by the progressive loss of upper and lower motor neurons in the spinal cord, cerebral cortex, and brainstem, resulting in muscle weakness and wasting (7, 8). Life expectancy is 2–5 years after onset (9). Approximately 5% of ALS patients develop frontotemporal dementia (FTD) and the ALS-FTD spectrum is hereafter referred to as ALS (10). Around 90% of ALS cases are sporadic (sALS) and 10% are familial (fALS), being associated with inherited mutations. Multiple genes have been linked with ALS (11). Interestingly, some ALS-associated genes, including *TARDBP* and *FUS*, encode RNA-binding proteins which are involved in miRNA processing (12), and indirectly implicate miRNAs in ALS pathophysiology. However in addition to RNA metabolism, ALS-associated genes show diverse functions, with roles in intracellular transport, proteostasis, axonal outgrowth, and glutamatergic signaling (7).

MiRNAs are unusually well-preserved in a range of biological samples, including blood plasma, serum, and cerebrospinal fluid, and are measurable with greater sensitivity and stability than proteins (5, 13). As a result, the last decade has seen a drive to identify specific miRNA biomarkers for ALS, in order to potentiate more rapid and accurate diagnosis, disease stratification and monitoring. Numerous studies have demonstrated deregulation of miRNAs in ALS patients, most aiming to identify clinically-relevant biomarkers.

Relevant to the ALS context, CSF miRNAs are potentially good representatives of central nervous system (CNS) disorders, since a blood-CSF barrier would prevent CNS miRNA dilution in the wider circulation (14). However, it is possible for miRNAs to transfer across this barrier, such that blood miRNAs may provide a window on nervous system dysfunction (15). Although the functional significance of circulating miRNAs is less clear, it has been demonstrated that cells can transfer functional miRNAs between one another in an exosome-mediated manner (16). It has been proposed cells can select the miRNAs to be released (17), although cells also shed material when degenerating. Thus, extracellular vesicles (EVs) may reflect the cells of origin, and some of these circulating miRNAs potentially mirror ALS pathophysiology.

Despite considerable efforts, no specific, robust diagnostic molecular biomarker set has been identified for ALS (18). Recently, Dardiotis et al. (19) reviewed the results of 24 studies, from 2010 to 2017, documenting miRNAs reported in ALS biomarker studies, aiming to clarify those most appropriate for future evaluation. In this same Frontiers issue, Joilin et al. (20) review recent attempts to define a “biomarker-relevant” signature of miRNAs, discussing their great potential and the challenges once the field moves toward clinical validation. However, beyond the key importance of biomarker identification, most studies so far do not attempt systematic bioinformatic or experimental functional interpretation of transcripts targeted by ALS-relevant miRNAs. Consequently, whether changes in miRNA expression simply reflect cellular dysfunction and degeneration, or are active participants in the functional changes of target genes relevant to disease pathogenesis, is unknown.

Here, we also focus on miRNA profiling studies comparing expression levels of miRNAs from ALS patients and controls, over the 2013–2018 period. Our approach aims to evaluate various strategies that can be used to analyse these deregulated miRNAs: *number of reported studies for a given miRNA, predicted functional targets*, and *tissue distribution* (i.e., where detected). We document the overlap between miRNAs reported as deregulated in these studies; and for these miRNAs, propose a series of *in silico* methods to identify those predicted to target known ALS genes, evaluating current limitations of such predictions in informing disease pathogenesis. Finally, we consider the source of patient tissue samples used for miRNA profiling, highlighting overlap of given miRNAs and revealing the importance of sample analyzed.

## Results and Discussion

### Literature Analysis

To define relevant studies we performed a PubMed literature search with the MeSH terms “microRNA” AND “amyotrophic lateral sclerosis” from 1/1/2013-31/12/2018. We identified 27 peer-reviewed studies fulfilling our selection criteria, which specifically included those recording and comparing levels of multiple miRNAs directly from ALS patients and controls (Table S1). Of these studies, 15 were previously considered by Dardiotis and colleagues, whilst Joilin et al. (20) in this same issue considered 11 of the studies presented here.

Detailed observation noted a large degree of variation between the studies, from sample source (serum, plasma, whole blood, CSF, spinal cord, muscle etc.), numbers and clinical characteristics of patient participants (both sALS and fALS) and controls (healthy and other diseases), to the methods used for sample preparation, miRNA profiling and analysis. Additionally, we identified the need for reporting specific arms of mature miRNAs, since in ambiguous cases we could only assume the dominant strand as that reported/detected (miRBase release 22.1: 2018).

In those few studies that investigate functional implications potentially derived from miRNA changes, a wide variety of bioinformatic approaches were used to identify possible mRNA targets of deregulated miRNAs, including different versions of TargetScan, Pictar, miRanda, DIANA-Tarbase, and miRtarbase. Further attempts to identify those gene/signaling networks targeted, built on protein-protein interaction (PPI) networks, gene ontology and pathway analysis, generating a variety of outcomes (21, 22).

### Most Commonly Deregulated microRNAs

As a first approach to select potentially pathologically relevant miRNAs, we ranked them according to the number of times they were reported as deregulated in different studies. In the 27 miRNA profiling studies, a total of 559 miRNAs were shown as deregulated. Among these, nine miRNAs were reported six or more times, compared to 38 reported in five or more studies, directing the threshold selected for our analysis. Those nine most frequently reported (≥6) are shown in Table 1A. Since any miRNA deregulation could have deleterious effects on gene targets, initial selection did not discriminate between up- vs. down-regulation. Indeed, for many miRNAs, the reported direction of deregulation was inconsistent between studies, which may be accounted for by differences in the analytical protocol and/or miRNA profiling technique.

**Table 1:** The ALS genes predicted by DIANA-microT-CDS v5.0 to be targets of (A) the nine most frequently reported miRNAs from the studies, (B) nine deregulated miRNAs randomly selected from all ALS studies, and (C) nine randomly selected miRNAs not reported to be deregulated in the ALS studies.

	**miRNAs**	**Number of Studies deregulated (out of 27)**	**Direction of deregulation (up, down or both)**	**ALS genes predicted by DIANA-T-CDS**						
(A) Most Frequently Reported miRNAs	hsa-miR-133a-3p	9	Both	*TUBA4A*	*VAPB*					**MiRNA hits (8/9) Genes (15/37) Total (18)**
	hsa-let-7a-5p	7	Both	*ARHGEF28*						
	hsa-miR-127-3p	6	Both							
	hsa-miR-155-5p	6	Both	*TBK1*	***UBQLN2***					
	hsa-miR-206-3p	6	Both	*ATXN2*	***MATR3***					
	hsa-miR-26a-5p	6	Both	*ARHGEF28*	*ERBB4*	*MATR3*				
	hsa-miR-455-3p	6	Both	*TARDBP*						
	hsa-miR-9-5p	6	Both	***CHMP2B***	*CRYM*	*NEFH*	***TRPM7***			
	hsa-miR-124-3p	6	Both	***CHMP2B***	*SIGMAR1*	*SQSTM1*				
(B) Random miRNAs from ALS studies	hsa-let-7b-5p	5	Down	*ARHGEF28*						**MiRNA hits (7/9) Genes (10/37) Total (16)**
	hsa-let-7c-5p	4	Down	*ARHGEF28*						
	hsa-miR-204-3p	1	Down	*ERBB4*	*VAPB*					
	hsa-miR-766-3p	2	Both	***DAO***	*DCTN1*	*SIGMAR1*	*VAPB*			
	hsa-miR-212-3p	2	Down	*C9orf72*	*ERBB4*	*FIG4*				
	hsa-miR-329-3p	2	Down							
	hsa-miR-876-3p	1	Down	*ERBB4*	*MATR3*	*TUBA4A*				
	hsa-miR-302a-5p	1	Down	***ARHGEF28***	*ERBB4*					
	hsa-miR-154-5p	3	Down							
(C) Random miRNAs absent from ALS studies	hsa-miR-3168-5p			*TARDPB*						**MiRNA hits (5/9) Genes (8/37) Total (13)**
	hsa-miR-875-5p									
	hsa-miR-611-5p									
	hsa-miR-603-3p			*ATXN2*	*ERBB4*	*MATR3*	*OPTN*	*TARDBP*	***VAPB***	
	hsa-miR-500b-5p			*DCTN1*	*ERBB4*					
	hsa-miR-325-5p									
	hsa-miR-764-5p									
	hsa-miR-665-3p			*DCTN1*						
	hsa-miR-4277-5p			*DAO*	*TARDPB*	*VAPB*				

*Note that we report mature miRNAs; where specific miRNAs were not reported we considered dominant strands as reported on miRBase release 22.1: October 2018. For (A), all miRNAs deregulated in at least six studies were considered. For (B) nine miRNAs were randomly selected from those reported by <6 of the 27 studies. Further analysis regarding strand specificity was also performed on this group and on the nine miRNAs not found deregulated in the studies (C). MiRNAs in bold have confirmed interactions with the target experimentally, using miRTarBase v7.0 (23)*.

Of the most frequently reported miRNAs, hsa-miR-133a-3p was found deregulated in 9/27 studies. The high ranking of hsa-miR-133a-3p may be explained by the fact it is a known myomiR, enriched in muscle tissue (24) and several of the analyzed studies focused on expression levels of myomiRs alone, potentially introducing tissue bias (25–29) (Table S1). However despite its myomiR label, hsa-miR-133a-3p has also been suggested as motor neuron enriched (30).

### Frequently Deregulated microRNAs and Target Prediction of ALS Genes

To connect biomarker reporting and potential functional relevance we have outlined an *in silico* method to determine whether these commonly reported miRNAs preferentially target selected known ALS-associated genes (http://alsod.iop.kcl.ac.uk/ [last updated 2015], an ALS bioinformatics repository online database) (31). The 37 ALS genes considered were (in alphabetical order):

*ALS2, ANG, ARHGEF28, ATXN2, C9orf72, CHCHD10, CHGB, CHMP2B, CRYM, DAO, DCTN1, ERBB4, FIG4, FUS, GLE1, LUM, MATR3, NEFH, OPTN, PARK7, PFN1, PLEKHG5, SETX, SIGMAR1, SOD1, SPG11, SQSTM1, SS18L1, SYNE, TAF15, TARDBP, TBK1, TRPM7, TUBA4A, UBQLN2, VAPB*, and *VCP*.

Although not updated since 2015, this database provides information regarding the ALS patients harboring mutations in these genes, such as patient numbers (fALS and sALS), gender and mean onset age as well as site of disease (bulbar/limb). Additionally, all ALS genes reviewed by Kirby et al. (10) except *hnRNPA1* are included in this list. For the prediction analysis we used DIANA-microT-CDS v5.0 (32, 33). As reviewed by Riffo-Campos et al. (34), the DIANA-microT attempts to apply a more balanced predictive approach, displaying TargetScan, and miRanda comparisons in its analysis.

From the *in silico* analysis, 8/9 most frequently deregulated miRNAs were predicted to target at least one of these ALS genes (Table 1A), with hsa-miR-9-5p, predicted to target 4/37 of the genes. There appeared to be no obvious relationship between the total number of ALS genes the individual miRNAs were predicted to target and the number of studies reporting these miRNAs as deregulated. The most frequently predicted ALS targeted genes were *ARHGEF28, CHMP2B*, and *MATR3* (2/9 miRNAs). The total count of predicted ALS target genes for the combined nine miRNAs was 18, and overall, 15/37 ALS genes were predicted as targets of at least one of the nine miRNAs. Whilst this approach highlights the potential of *in silico* predictive methods, evaluation of comparable analyses with less frequently reported deregulated miRNAs is also merited.

### Other Deregulated microRNAs and Target Prediction of ALS Genes

To determine if the most commonly identified ALS miRNAs are the most relevant, the same analysis must be performed with an identical number (nine) of randomly selected miRNAs, which although reported to be deregulated in the 27 ALS studies, appeared in fewer than six reports. Randomization was achieved by selecting from all deregulated miRNAs, without duplicates, using a Microsoft Excel randomization function. The results of this preliminary analysis are shown in Table 1B. Of the nine miRNAs, deregulation was reported in between 1 and 5 (of 27) studies. 7/9 of these randomly selected miRNAs were predicted to target at least one of the 37 ALS genes. Further, one of the miRNAs, hsa-miR-766-3p (deregulated in 2/27 studies) was predicted to target 4/37 ALS genes. The most frequently predicted ALS targeted gene was *ERBB4* (4/9 miRNAs). The total count of predicted ALS target genes for the combined nine miRNAs was 16, comparable to that of the nine most commonly deregulated miRNAs (18, Table 1A). Overall, 10/37 ALS genes were predicted as targets of at least one of the nine miRNAs. Although this second analysis could indicate all deregulated miRNAs are equally important in their capacity to potentially target ALS relevant genes, a further *in silico* step requires comparison with a group of miRNAs not deregulated in biomarker studies.

### Analysis of Randomly Selected and Non-deregulated microRNAs

To investigate if predicted gene targets for deregulated ALS miRNAs reflected an enrichment compared to non-deregulated miRNAs, we performed an example test with nine randomly selected mature miRNAs not reported as deregulated in any of the 27 studies and performed the same analysis (Table 1C). We selected from all *Homo sapien* mature miRNA sequences recorded on miRBase release 22.1: October 2018 (35). One of the random miRNAs, hsa-miR-603-3p, was predicted to target 6/37 of the ALS genes, including *ERBB4*. The major ALS gene, *TARDBP*, was predicted most frequently (3/9 miRNAs).

Compared to the nine most frequently reported (Table 1A) or not frequently reported deregulated miRNAs (Table 1B), this random miRNA selection gave a total count of 13 predicted ALS target genes (Table 1C), representing only 8/37 of the ALS genes. Notably, 5/9 of these random miRNAs were predicted to target at least one of the 37 ALS genes. Further selections of different sets of nine random miRNAs showed similar results (not shown).

The systematic approach outlined above would potentially allow the use of statistical analysis (i.e., binomial test) to indicate whether deregulated miRNAs from ALS patient studies more frequently target ALS genes (at least based on *in silico* predictions) than randomly selected miRNAs, but we suggest greater numbers of miRNAs would need to be considered. This analysis would not be trivial and is outside the scope of this article, which seeks to propose a workflow. Further, consideration of additional (to the 37 used here) ALS genes is likely merited, again expanding the complexity of the analysis. Current ALS genes also relate to different signaling networks, and more specific gene pathways may be required for target enrichment. In summary, we have defined a systematic *in silico* analysis that should be extended in the future to investigate functional links between deregulated miRNAs and ALS pathological processes.

### Tissue Distribution of Deregulated miRNAs

**Figure 1:**
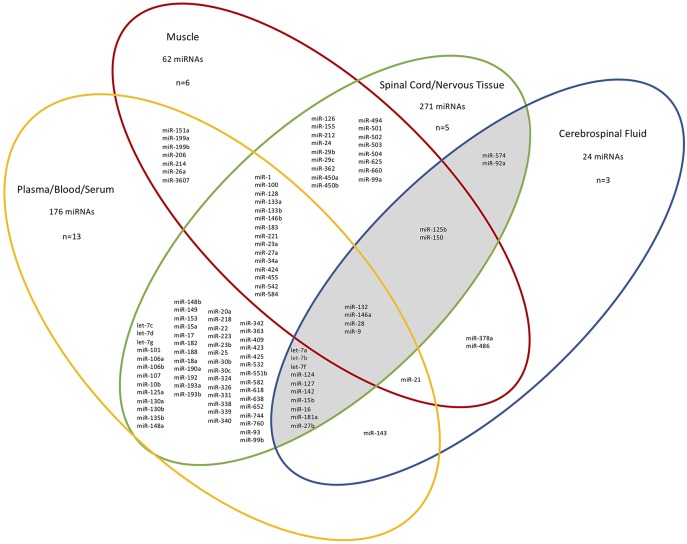
Different tissue sources and overlap of miRNAs identified from ALS patients in 26/27 studies. n=the number of papers examined in each compartment. For simplicity, specific miRNA arms are not shown. The CSF-spinal cord/nervous tissue overlap is shaded. The miRNAs deregulated between patient neuromuscular junction and control blood and deregulated miRNAs from sALS patient fibroblasts are not included since they do not belong in any of the distinct source group compartments used here (21, 36). The total miRNAs present in each group are given. MiRNAs within a single compartment are not shown.

Next, we considered the different tissue sources of the ALS-relevant miRNAs identified in 26/27 studies (*n* = 410 unique miRNAs, for exclusions see Figure 1 legend), and grouped the miRNAs based on four “compartments”: CSF, spinal cord/nervous tissue, muscle, and plasma/blood/serum. Figure 1 shows the overlap between studies after mapping the deregulated miRNAs to the compartment (tissue) they were determined in. 265/410 deregulated miRNAs were present in at least two different tissues. We noted considerable correspondence between miRNAs extracted from plasma/blood/serum with those from CSF (total of 16/24 miRNAs within the CSF group), supporting the notion that blood miRNAs can provide a window into CSF changes (15). Since ALS is a neurodegenerative disorder, those miRNAs deregulated in patient CSF and spinal cord/nervous tissue were of particular interest and these 18 miRNAs are shown in a shaded region in Figure 1. Of these 18 miRNAs, two are exclusive to only these two sources (hsa-miR-92a-5p/3p and hsa-miR-574-5p/3p). Notably, of the nine most frequently reported miRNAs found to be deregulated in the 27 studies (Table 1A), four overlapped with these 18 miRNAs.

### Most Frequently Deregulated miRNAs Within the CSF-Spinal Cord/Nervous Tissue Overlap

As noted above, four miRNAs in the CSF-spinal cord/nervous tissue overlap are also amongst the most frequently reported deregulated miRNAs (Table 1A; hsa-miR-124-3p, hsa-miR-127-3p, hsa-let-7a-5p, and hsa-miR-9-5p). The latter is discussed in the section MicroRNAs Present in all Tissue Sources.

Hsa-miR-124-3p was reported down-regulated in ALS patients in five studies (21, 37–40), with only one study finding it upregulated (41). Despite the caveats to the predictive approach highlighted above, it is notable that the predicted targets of this miRNA are *CHMP2B, SQSTM1*, and *SIGMAR1*. Hsa-miR-124-3p has been shown to be deregulated in the spinal cord and brainstem of *SOD1* transgenic mice and has been linked to astrocyte differentiation and neurogenesis in the mouse brain (42, 43). Further, hsa-miR-124-3p is found to be expressed almost exclusively in the brain and spinal cord (44).

Hsa-miR-127-3p was not predicted to target any of the ALS genes and was found almost consistently down-regulated in ALS patients (22, 37, 39, 45, 46) with only one study reporting its upregulation (41). Whilst little is reported in relation to ALS, hsa-miR-127-3p has been found deregulated in FTD patients compared to control groups and Alzheimer's disease patients (47). This result is consistent with hsa-miR-127-3p predominantly being expressed in brain tissue (44).

Hsa-let-7a-5p, most highly expressed in the cerebellum, is predicted to target *ARHGEF28* (Table 1A) (44). Let-7a-5p has been found downregulated in the plasma of Parkinson's disease patients compared to healthy controls, showing it may not be useful as an ALS specific biomarker (48).

### MicroRNAs Unique to the CSF-Spinal Cord/Nervous Tissue Overlap

The two miRNAs unique to the CSF-spinal cord/nervous tissue group are hsa-miR-92a-5p/3p and hsa-miR-574-5p/3p. Hsa-miR-92a-5p/3p's predicted targets are *CHCHD10, TARDBP, PLEKHG5*, and *NEFH* and hsa-miR-574-5p/3p's are *VAPB* and *SIGMAR1*. According to a miRNA tissue atlas, both miRNAs show neither specific tissue specificity nor ubiquitous expression (44). Despite this, deregulation of these miRNAs in ALS could be tissue specific.

### MicroRNAs Present in all Tissue Sources

Hsa-miR-132-5p/3p, hsa-miR-146a-5p/3p, hsa-miR-28-5p/3p, and hsa-miR-9-5p/3p were deregulated in all tissue samples (Figure 1) and are all predicted to target at least one ALS gene. Hsa-miR-132-3p has been implicated in a range of neurodegenerative disorders including multiple sclerosis, Parkinson's disease and Alzheimer's disease, demonstrating wider relevance beyond ALS (49). This is consistent with the miRNA tissue atlas, where it is primarily expressed in the brain (44). Downregulation of miR-146a-5p in cortical aberrant astrocytes has been implicated in motor neuron degeneration in ALS, whereas its upregulation has been implicated in motor neuron loss in spinal muscular atrophy (50, 51). No links between miR-28-5p/3p and ALS have yet been made, consistent with it being predicted to target just one ALS-associated gene (*SETX*). Mutations in *TARDBP* have been reported to cause deregulation of miR-9-5p and miR-9-5p/3p has been shown to be upregulated in mutant *SOD1* mice (52, 53). MiR-9-5p has been implicated in axon extension and branching *via* targeting of *Map1b* (54). It is also predominantly expressed in the brain and spinal cord (44).

## Conclusions

We have shown that miRNAs found deregulated in published studies investigating ALS patients have limited overlap, likely due to the wide variation in tissue extraction and miRNA detection methods. Future emphasis should therefore be on standardizing tissue extraction and miRNA profiling methods.

However, we identified nine miRNAs repeatedly reported as deregulated in the 27 studies. Despite these miRNAs being commonly predicted to target ALS-associated genes, the randomly selected miRNAs not found deregulated in ALS patients, showed similar predictions. Therefore, our *in silico* analysis provided no clear correlation between deregulated miRNAs and the collection of ALS-linked genes analyzed. This indicates that whilst the ability to predict thousands of candidate genes with *in silico* methods remains informative, they should be used with caution and in combination with other methods, of which experimental functional testing is recommended. Although limitations of the bioinformatics approach may explain our observations, the currently identified ALS-associated genes may offer a limited view on the pathological pathways altered during disease progression. It is thus tempting to suggest *in silico* analyses are currently underpowered. In the future it would be interesting to perform this bioinformatics approach using ALS genes grouped by their relation to specific functional pathways, for example proteostasis or RNA metabolism.

We have additionally shown the source can influence the miRNAs detected, since only four deregulated miRNAs appeared in all tissue sources analyzed. Importantly, we have shown the four miRNAs reported most frequently deregulated appear in CSF, spinal cord/nervous tissue and blood/plasma/serum. This suggests miRNAs may indeed “travel” between CSF and blood, the latter potentially providing a clinically accessible source which may mirror ALS pathology in the CNS. We therefore propose four miRNAs—hsa-miR-124-3p, hsa-miR-127-3p, hsa-let-7a-5p, and hsa-miR-9-5p—as good candidates for further study and suggest blood, serum or plasma as a clinically accessible source.

Overall we have demonstrated the need for a multifaceted approach, utilizing patient data, bioinformatics, but most critically, experimental follow-up, to resolve the true biological significance of these implicated miRNAs and determine the real disease culprits of ALS.

### Note Added After Submission

Whilst this manuscript was under review, Yao et al. (55) identified *SQSTM1* as a target of miR-124-3p. Notably our analysis had indicated that hsa-miR-124-3p, present in the CSF-spinal cord/nervous tissue overlap (Figure 1), was one of the most frequently reported deregulated miRNAs (6/27 studies) and was predicted to target *SQSTM1* (Table 1A).

## Author Contributions

SF wrote and edited the manuscript. RL, FD-B, and RM-R critically reviewed and edited the manuscript.

### Conflict of Interest Statement

The authors declare that the research was conducted in the absence of any commercial or financial relationships that could be construed as a potential conflict of interest.
