# Supplementary material for: Biological Significance of microRNA Biomarkers in ALS—Innocent Bystanders or Disease Culprits?
Source: Front Neurol. 2019 Jun 11;10:578. doi: 10.3389/fneur.2019.00578 (PMC6579821; doi:10.3389/fneur.2019.00578)
Supplement: Supplementary file 1 [file Table_1.docx]

Table S1: Features of studies comparing miRNA levels in ALS patients and healthy controls. Studies are organised chronologically, with newest first. Note both familial and sporadic ALS patients are used in some studies and the variation in the samples obtained from patients. Also note the variation in the techniques used for miRNA detection or quantification, and functional or bioinformatic assessments. HCs=healthy controls.

| Study | Sample | Participants | Main Methods and Techniques |
| --- | --- | --- | --- |
| Saucier et al., 2018 | Extracellular vesicles from plasma | 14 ALS patients and 12 HCs; NGS.  12 ALS and 3 HCs; quantification. | miRNA expression levels identified through next generation sequencing (NGS). Quantification performed using reverse transcription and digital droplet PCR.  Prediction of gene targets performed by TargetScan 7.1 and human miRanda algorithm. Functional relevance assessed with DAVID. |
| Taguchi and Wang 2018 | Serum | 9 fALS, 18 sALS, 18 ALS mutation carriers and 17 HCs. | ﻿miRNA expression profiles produced by Freidschmidt et al. 2014, were subject to principal component analysis – based unsupervised feature extraction. KEGG pathway enrichment analysis performed on the downregulated miRNAs. |
| Kovanda et al., 2018 | Muscle biopsies | 11 ALS patients and 11 HCs. | NGS was followed by differential expression analysis of miRNAs. miRNA analysis performed using Tarbase and KEGG and Gene ontology (GO) analysis performed. |
| Raheja et al., 2018 | Serum | 20 sALS patients, 3 fALS patients and 30 HCs. | miRNA detection performed using RT-PCR. |
| Liguori et al., 2018 | Peripheral blood | 6 sALS and 5 HCs; discovery phase.  50 sALS patients and 15 HCs; validation. | High- throughput NGS and a subset validated using qRT-qPCR. Gene targets were predicted using miRtarbase and DIANA-Tarbase and pathway analysis was performed using DAVID. |
| De Felice et al., 2018 | Neuromuscular junction and blood leukocytes | 45 sALS patients and 25 HCs. | Samples analysed by next generation sequencing and a selection were validated by qRT-PCR. Target prediction performed using TargetScan and Pictar. KEGG and GO analysis were performed. |
| D’Erchia et al., 2017 | Ventral horns of the lumbar spinal cord from post mortems | 11 sALS and 7 HCs. | Transcriptomes sequenced with RNA-seq and validated with qRT-PCR. Differentially expressed genes identified using CuffDiff2 and DEseq2. Ingenuity pathway and GO analysis performed. |
| Waller et al., 2017a | CSF | 32 sALS patients and 10 HCs with 6 neurological controls (MS patients) combined. | qPCR and small RNA sequencing and qPCR was used in validation. The online platforms Oasis 2.0 (Capece et al., 2015) and Genboree (Subramanian et al., 2015) were used to analyse the small RNA sequencing data. |
| Waller et al., 2017b | Serum | 27 sALS patients, and 25 HCs.  23 sALS patients and 22 HCs; validation. | The expression levels of 750 miRNAs were determined using Taqman arrays and qPCR was performed on these miRNAs. Validation qPCR was performed on a selection of 27 miRNAs. |
| Di Pietro et al., 2017 | Skeletal muscle | 14 ALS patients and 24 HCs. | RNA isolated using TaqMan microRNA Reverse Transcription kit and quantified using specific probes. Functional characterisation of one miRNA performed in cells. |
| Pegoraro et al., 2017 | Skeletal muscle | 13 sALS patients and 5 HCs. | qRT-PCR performed on extracted miRNA. |
| Figueroa-Romero et al., 2016 | Spinal cord from post mortems | 12 sALS and 12 HCs. | TaqMan OpenArray miRNA profiling was performed and differential expression was confirmed with qPCR. Validation performed by qPCR. Validated miRNA targets obtained from TarBase and predicted targets from TargetScan and microrna.org |
| Benigni et al., 2016 | CSF | 24 sALS patients and 24 HCs. | Quantitative reverse transcription PCR identified deregulated miRNAs. MiRNA profiling was performed by qRT-PCR and validated by RT-PCR. |
| Jensen et al., 2016 | Skeletal muscle | 5 ALS patients and 7 HCs. | Reverse transcription performed using TaqMan MicroRNA Reverse Transcription Kit. MiRNA analysis performed using specific primers for skeletal muscle specific miRNAs. |
| Tasca et al., 2016 | Muscle specific miRNAs in serum | 14 sALS patients and 8 HCs. | Expression levels quantified using qRT-PCR. |
| Chen et al., 2016 | Leukocytes | 5 sALS patients and 5 HCs.  83 sALS patients with 61 HCs; validation. | Microarray analysis identified differentially expressed miRNAs and validation was performed on all these miRNAs by RT-PCR. Target genes were predicted using TargetScan, PicTar, miRanda, PITA and RNA22. GO analysis was also performed. |
| de Andrade et al., 2016 | Skeletal muscle and plasma | 5 sALS and 5 HCs; skeletal muscle expression analysis:  39 sALS and 39 HC; plasma expression analysis. | Microarray analysis identified differentially expressed miRNAs from ALS patient’s muscle. Three miRNAs were validated in RT-qPCR in muscle. Targets predicted using miRGen algorithm. |
| Raman et al., 2015 | Fibroblasts | 6 sALS, 6 PLS (primary lateral sclerosis) and 6 HCs.  11 ALS, 6 PLS and 10 HCs; validation. | Microarray analysis identified differentially expressed miRNAs. DAVID and GO analysis were applied to differentially expressed transcripts. Validation performed using qRT-PCR. |
| Emde et al., 2015 | Motor neurons of spinal cords | 8 sALS, 9 non-neurodegeneration controls. | Taqman microarray and qPCR identified 667 miRNAs. RT-qPCR used to assess expression changes. |
| Takahashi et al., 2015 | Plasma | 16 sALS patients and 10 HCs; microarray cohort.  48 sALS patients and 30 HCs; validation cohort. | 3D-Gene microarray analysis using around 1800 probes identified differentially expressed miRNAs, some which were validated in qRT-PCR. |
| Freischmidt et al., 2015 | Serum | 18 sALS patients and 16 HCs.  20 sALS patients and 20 HCs; validation cohort:  13 fALS and 13 HCs; comparison group. | Microarray analysis with a focus on only downregulated miRNAs and validation was performed with qRT-PCR. |
| Freischmidt et al., 2014 | Serum | Group 1: 9 fALS and 10 HCs.  13 fALS patients with 13 HCs; validation group:  14 sALS patients with 14 HCs; second validation group. | Microarray analysis measured the abundance of 1733 mature miRNAs. Validation of 4 miRNAs using qRT-PCR. A second validation was performed with the same 4 miRNAs by qRT-PCR. DREME tool used to identify two motifs frequently occurring in the downregulated miRNAs of fALS and ALS mutation carrying patients. |
| Wakabayashi et al., 2014 | Formalin-fixed paraffin -embedded post mortem brain specimens | 6 ALS patients and 4 HCs. | Microarray analysis performed on extracted RNA and analysed for differential expression. Bioinformatic predictions of target genes performed using miRmap. Ontology analysis performed using Metacore functional analysis and gene ontology analysis performed using the Gene Ontology Consortium web tool. |
| Ishtiaq et al., 2014 | Ventral lumbar spinal cord | 5 sALS patients and 5 HCs. | Small RNAs were isolated and sequencing library was generated. Sequencing revealed 80 putative novel miRNAs and only those predicted to target NEFL mRNA were considered. Following structural analysis, RT-PCR confirmed 10 miRNAs were differentially expressed. Further analysis performed using functional reporter assays and relative quantitative PCR. |
| Campos-Melo et al., 2013 | Spinal cord | 5 sALS patients and 3 HCs. | Taqman microarrays used to identify 664 miRNAs and a selection of miRNAs were validated using RT-qPCR. Ingenuity pathway analysis performed. Bioinformatic analysis performed using TargetScan and microrna.org to identify miRNAs with recognition elements in the 3’UTR of NEFL. Functional analysis performed using reporter assays and RT-qPCR. |
| Freischmidt et al., 2013 | CSF and serum | 22 ALS patients and 24 HCs. | RNA isolated and the levels of TDP-43 binding miRNAs measured by qPCR. Confirmation performed in lymphoblast cell lines from genetically defined ALS patients. |
| Russell et al., 2013 | Skeletal muscle | 14 ALS patients and 10 HCs. | Specific skeletal muscle enriched miRNAs reversed transcribed using specific primers and levels measured using specific primers and probes. |

**References**

Benigni, M., Ricci, C., Jones, A. R., Giannini, F., Al-Chalabi, A., & Battistini, S. (2016). Identification of miRNAs as Potential Biomarkers in Cerebrospinal Fluid from Amyotrophic Lateral Sclerosis Patients. *NeuroMolecular Medicine*, *18*(4), 551–560. https://doi.org/10.1007/s12017-016-8396-8

Campos-Melo, D., Droppelmann, C. A., He, Z., Volkening, K., & Strong, M. J. (2013). Altered microRNA expression profile in amyotrophic lateral sclerosis: a role in the regulation of NFL mRNA levels. *Molecular Brain*, *6*(1), 26. https://doi.org/10.1186/1756-6606-6-26

Capece, V., Garcia Vizcaino, J. C., Vidal, R., Rahman, R.-U., Pena Centeno, T., Shomroni, O et al., (2015). Oasis: online analysis of small RNA deep sequencing data. *Bioinformatics (Oxford, England)*, *31*(13), 2205–2207. https://doi.org/10.1093/bioinformatics/btv113

Chen, Y., Wei, Q., Chen, X., Li, C., Cao, B., Ou, R et al., (2016). Aberration of miRNAs Expression in Leukocytes from Sporadic Amyotrophic Lateral Sclerosis. *Frontiers in Molecular Neuroscience*, *9*, 69. https://doi.org/10.3389/fnmol.2016.00069

D’Erchia, A. M., Gallo, A., Manzari, C., Raho, S., Horner, D. S., Chiara, M et al., (2017). Massive transcriptome sequencing of human spinal cord tissues provides new insights into motor neuron degeneration in ALS. *Scientific Reports*, *7*(1), 10046. https://doi.org/10.1038/s41598-017-10488-7

de Andrade, H. M. T., de Albuquerque, M., Avansini, S. H., de S. Rocha, C., Dogini, D. B., Nucci, A et al., (2016). MicroRNAs-424 and 206 are potential prognostic markers in spinal onset amyotrophic lateral sclerosis. *Journal of the Neurological Sciences*, *368*, 19–24. https://doi.org/10.1016/j.jns.2016.06.046

De Felice, B., Manfellotto, F., Fiorentino, G., Annunziata, A., Biffali, E., Pannone, R et al., (2018). Wide-Ranging Analysis of MicroRNA Profiles in Sporadic Amyotrophic Lateral Sclerosis Using Next-Generation Sequencing. *Frontiers in Genetics*, *9*, 310. https://doi.org/10.3389/fgene.2018.00310

Di Pietro, L., Baranzini, M., Berardinelli, M. G., Lattanzi, W., Monforte, M., Tasca, G et al., (2017). Potential therapeutic targets for ALS: MIR206, MIR208b and MIR499 are modulated during disease progression in the skeletal muscle of patients. *Scientific Reports*, *7*(1), 9538. https://doi.org/10.1038/s41598-017-10161-z

Emde, A., Eitan, C., Liou, L.-L., Libby, R. T., Rivkin, N., Magen, I et al., (2015). Dysregulated miRNA biogenesis downstream of cellular stress and ALS-causing mutations: a new mechanism for ALS. *The EMBO Journal*, *34*(21), 2633–2651. https://doi.org/10.15252/embj.201490493

Figueroa-Romero, C., Hur, J., Lunn, J. S., Paez-Colasante, X., Bender, D. E., Yung, R et al., (2016). Expression of microRNAs in human post-mortem amyotrophic lateral sclerosis spinal cords provides insight into disease mechanisms. *Molecular and Cellular Neurosciences*, *71*, 34–45. https://doi.org/10.1016/j.mcn.2015.12.008

Freischmidt, A., Müller, K., Ludolph, A. C., & Weishaupt, J. H. (2013). *Systemic dysregulation of TDP-43 binding microRNAs in amyotrophic lateral sclerosis*. https://doi.org/10.1186/2051-5960-1-42

Freischmidt, A., Müller, K., Zondler, L., Weydt, P., Mayer, B., von Arnim, C. A. F et al., (2015). Serum microRNAs in sporadic amyotrophic lateral sclerosis. *Neurobiology of Aging*, *36*(9), 2660.e15-2660.e20. https://doi.org/10.1016/J.NEUROBIOLAGING.2015.06.003

Freischmidt, A., Müller, K., Zondler, L., Weydt, P., Volk, A. E., Božič, A. L et al., (2014). Serum microRNAs in patients with genetic amyotrophic lateral sclerosis and pre-manifest mutation carriers. *Brain*, *137*(11), 2938–2950. https://doi.org/10.1093/brain/awu249

Ishtiaq, M., Campos-Melo, D., Volkening, K., & Strong, M. J. (2014). Analysis of Novel NEFL mRNA Targeting microRNAs in Amyotrophic Lateral Sclerosis. *PLoS ONE*, *9*(1), e85653. https://doi.org/10.1371/journal.pone.0085653

Jensen, L., Jørgensen, L. H., Bech, R. D., Frandsen, U., & Schrøder, H. D. (2016). Skeletal Muscle Remodelling as a Function of Disease Progression in Amyotrophic Lateral Sclerosis. *BioMed Research International*, *2016*, 5930621. https://doi.org/10.1155/2016/5930621

Kovanda, A., Leonardis, L., Zidar, J., Koritnik, B., Dolenc-Groselj, L., Ristic Kovacic, S et al., (2018). Differential expression of microRNAs and other small RNAs in muscle tissue of patients with ALS and healthy age-matched controls. *Scientific Reports*, *8*(1), 5609. https://doi.org/10.1038/s41598-018-23139-2

Liguori, M., Nuzziello, N., Introna, A., Consiglio, A., Licciulli, F., D’Errico, E et al., (2018). Dysregulation of MicroRNAs and Target Genes Networks in Peripheral Blood of Patients With Sporadic Amyotrophic Lateral Sclerosis. *Frontiers in Molecular Neuroscience*, *11*, 288. https://doi.org/10.3389/fnmol.2018.00288

Pegoraro, V., Merico, A., & Angelini, C. (2017). Micro-RNAs in ALS muscle: Differences in gender, age at onset and disease duration. *Journal of the Neurological Sciences*, *380*, 58–63. https://doi.org/10.1016/J.JNS.2017.07.008

Raheja, R., Regev, K., Healy, B. C., Mazzola, M. A., Beynon, V., Von Glehn, F et al., (2018). Correlating serum micrornas and clinical parameters in amyotrophic lateral sclerosis. *Muscle & Nerve*, *58*(2), 261–269. https://doi.org/10.1002/mus.26106

Raman, R., Allen, S. P., Goodall, E. F., Kramer, S., Ponger, L.-L., Heath, P. R et al., (2015). Gene expression signatures in motor neurone disease fibroblasts reveal dysregulation of metabolism, hypoxia-response and RNA processing functions. *Neuropathology and Applied Neurobiology*, *41*(2), 201–226. https://doi.org/10.1111/nan.12147

Russell, A. P., Wada, S., Vergani, L., Hock, M. B., Lamon, S., Léger, B et al., (2013). Disruption of skeletal muscle mitochondrial network genes and miRNAs in amyotrophic lateral sclerosis. *Neurobiology of Disease*, *49*, 107–117. https://doi.org/10.1016/J.NBD.2012.08.015

Saucier, D., Wajnberg, G., Roy, J., Beauregard, A.-P., Chacko, S., Crapoulet, N et al., (2018). Identification of a circulating miRNA signature in extracellular vesicles collected from amyotrophic lateral sclerosis patients. *Brain Research*. https://doi.org/10.1016/J.BRAINRES.2018.12.016

Subramanian, S. L., Kitchen, R. R., Alexander, R., Carter, B. S., Cheung, K.-H., Laurent, L. C et al., (2015). Integration of extracellular RNA profiling data using metadata, biomedical ontologies and Linked Data technologies. *Journal of Extracellular Vesicles*, *4*, 27497. https://doi.org/10.3402/jev.v4.27497

Taguchi, Y.-H., & Wang, H. (2018). Exploring microRNA Biomarker for Amyotrophic Lateral Sclerosis. *International Journal of Molecular Sciences*, *19*(5). https://doi.org/10.3390/ijms19051318

Takahashi, I., Hama, Y., Matsushima, M., Hirotani, M., Kano, T., Hohzen, H et al., (2015). Identification of plasma microRNAs as a biomarker of sporadic Amyotrophic Lateral Sclerosis. *Molecular Brain*, *8*(1), 67. https://doi.org/10.1186/s13041-015-0161-7

Tasca, E., Pegoraro, V., Merico, A., & Angelini, C. (2016). Circulating microRNAs as biomarkers of muscle differentiation and atrophy in ALS. *Clinical Neuropathology*, *35*(01), 22–30. https://doi.org/10.5414/NP300889

Wakabayashi, K., Mori, F., Kakita, A., Takahashi, H., Utsumi, J., & Sasaki, H. (2014). Analysis of microRNA from archived formalin-fixed paraffin-embedded specimens of amyotrophic lateral sclerosis. *Acta Neuropathologica Communications*, *2*, 173. https://doi.org/10.1186/S40478-014-0173-Z

Waller, R., Goodall, E. F., Milo, M., Cooper-Knock, J., Da Costa, M., Hobson, E et al., (2017b). Serum miRNAs miR-206, 143-3p and 374b-5p as potential biomarkers for amyotrophic lateral sclerosis (ALS). *Neurobiology of Aging*, *55*, 123–131. https://doi.org/10.1016/J.NEUROBIOLAGING.2017.03.027

Waller, R., Wyles, M., Heath, P. R., Kazoka, M., Wollff, H., Shaw, P. J et al., (2017a). Small RNA Sequencing of Sporadic Amyotrophic Lateral Sclerosis Cerebrospinal Fluid Reveals Differentially Expressed miRNAs Related to Neural and Glial Activity. *Frontiers in Neuroscience*, *11*, 731. https://doi.org/10.3389/fnins.2017.00731
